# Family history of colorectal cancer and survival: a Swedish population‐based study

**DOI:** 10.1111/joim.13036

**Published:** 2020-03-03

**Authors:** F. Pesola, S. Eloranta, A. Martling, D. Saraste, K. E. Smedby

**Affiliations:** ^1^ School of Cancer & Pharmaceutical Sciences King's College London London UK; ^2^ Department of Medicine Solna Division of Clinical Epidemiology Karolinska Institutet and Karolinska University Hospital Stockholm Sweden; ^3^ Department of Molecular Medicine and Surgery Karolinska Institutet Stockholm Sweden

**Keywords:** colorectal cancer, family history, flexible parametric models, prognosis, survival

## Abstract

**Objectives:**

A family history of colorectal cancer (CRC) is an established risk factor for developing CRC, whilst the impact of family history on prognosis is unclear. The present study assessed the association between family history and prognosis and, based on current evidence, explored whether this association was modified by age at diagnosis.

**Methods:**

Using data from the Swedish Colorectal Cancer Registry (SCRCR) linked with the Multigeneration Register and the National Cancer Register, we identified 31 801 patients with a CRC diagnosed between 2007 and 2016. The SCRCR is a clinically rich database which includes information on the cancer stage, grade, location, treatment, complications and postoperative follow‐up.

**Results:**

We estimated excess mortality rate ratios (EMRR) for relative survival and hazard ratios (HR) for disease‐free survival with 95% confidence intervals (CIs) using flexible parametric models. We found no association between family history and relative survival (EMRR = 0.96, 95% CIs: 0.89–1.03, *P* = 0.21) or disease‐free survival (HR = 0.98, 95% CIs: 0.91–1.06, *P* = 0.64). However, age was found to modify the impact of family history on prognosis. Young patients (<50 at diagnosis) with a positive family history had less advanced (i.e. stages I and II) cancers than those with no family history (OR = 0.71, 95% CI: 0.56–0.89, *P* = 0.004) and lower excess mortality even after adjusting for cancer stage (EMMR = 0.63, 95% CIs: 0.47–0.84, *P* = 0.002).

**Conclusions:**

Our results suggest that young individuals with a family history of CRC may have greater health awareness, attend opportunistic screening and adopt lifestyle changes, leading to earlier diagnosis and better prognosis.

## Graphical abstract

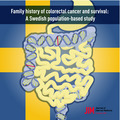

## Introduction

Colorectal cancer (CRC) is the third most common cancer and the second leading cause of cancer death in the world 1. Survival has been increasing over the years, and prognosis is associated with age at diagnosis, cancer stage, grade as well as treatment in both men and women 2. Evidence indicates there is a heritable component of CRC, which has been previously estimated to be between 15% and 30% 3, 4 with approximately 5% of patients having highly penetrant, hereditary syndromes (e.g. Lynch syndrome and familial adenomatous polyposis) 5.

Individuals with an affected first‐degree relative (i.e. positive family history) have a 2‐ to 4‐fold risk of developing cancer compared to individuals with a negative family history 6; however, the association between family history and prognosis is not as well established. There is extensive evidence showing improved survival in patients diagnosed with hereditary nonpolyposis CRC (e.g. Lynch syndrome) due to both surveillance and cancer characteristics 7, 8. Nonetheless, there is mixed evidence for the association between familial risk and survival beyond highly penetrant syndromes. A small number of studies found no association between CRC family history and prognosis 9, 10, 11. Some studies observed worsened 12, 13, whilst others showed improved 14, 15, 16, 17, 18, 19 prognosis amongst patients with a positive (vs. negative) family history. In a few of these studies, the favourable prognosis observed amongst patients with a positive CRC family history appeared to be present for colon but not rectum cancers 15, 17, 19. However, the modifying role of location was not systematically assessed in these studies.

Even though age at diagnosis has been found to be positively associated with CRC prognosis, with young patients having better prognosis than their older counterparts despite worse staging 20, 21, 22, few studies have explored the interplay between age at diagnosis and family history. A retrospective study using the Utah Cancer Registry data observed that young (age <56) men with a sibling with CRC had worse prognosis than their young counterparts with no family history 16, whilst another study found no evidence of an interaction between age at diagnosis (<60 vs. 60+) and self‐reported family history 11.

Using Swedish population‐based register data, we assessed potential differences in clinical characteristics as well as prognostic differences (i.e. relative survival and disease‐free survival) in CRC patients with positive and negative family histories. Moreover, based on previous findings, we aimed to explore whether age at diagnosis or cancer location (i.e. colon vs. rectal cancer) modified the effect of family history on survival.

## Methods

### Data sources

The study cohort was obtained from the Swedish Colorectal Cancer Registry (SCRCR). This register collects information on all patients diagnosed with adenocarcinoma of the colon and rectum in Sweden since 1995 for rectal cancer and 2007 for colon cancer. It contains information regarding patients and tumour characteristics, preoperative staging and investigations, perioperative details, oncological treatment and postoperative 5‐year follow‐up. This register has been found to have good validity 23 and to capture 99% of all patients diagnosed with CRC in Sweden 24.

To identify relatives with a CRC diagnosis, we linked the SCRCR with the Multigeneration Register and the Swedish Cancer Register. The Multigeneration Register contains information for all individuals born in Sweden from 1932 and alive in 1961. This register has a high coverage from 1991, whereas earlier information is not complete (i.e. left truncation) 25, 26. The Swedish Cancer Register was established in 1958 and covers all of Sweden. This register codes all cancer cases using the International Classification of Disease version 7 (ICD‐7) with recent versions also available for current years. This register has good national coverage with less than 4% under‐reporting 27. Vital status was obtained from the Cause of Death Register and emigration information, to be used for censoring, from the Total Population Register.

### Study population

**Figure 1:**
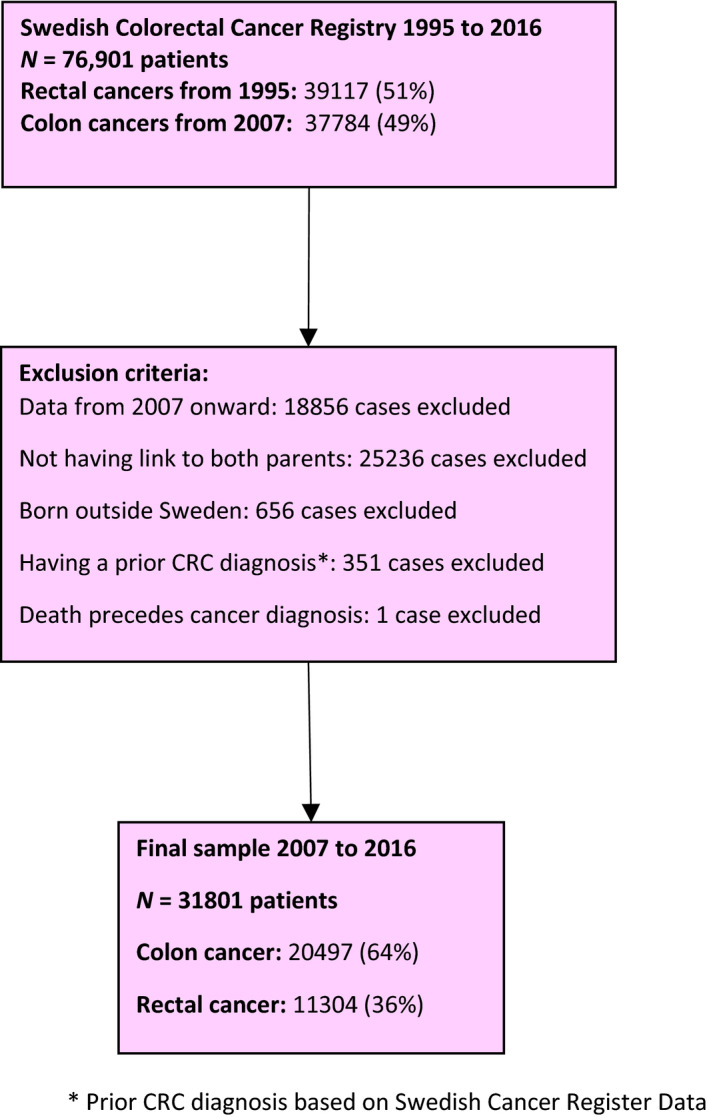
Flow chart summarizing how the final study population was obtained: 31 801 patients with a primary colorectal cancer diagnosed in Sweden between 2007 and 2016.

Due to the fact that the SCRCR started recording colon cancers in 2007, analyses were restricted to years 2007 onwards. In line with previous studies, we were interested in including patients for whom both parents could be identified in the Multigeneration Register 9, 28. Due to potential left truncation in the Multigeneration Register, only patients born after 1932 were included in the analyses 25, 26. Additionally, the register has been found to be less complete for individuals born outside of Sweden 26 and, therefore, we only included Swedish‐born patients. Even after applying these exclusion criteria, it was not possible to identify both parents for 5745 patients (of 37 688, 15%) and, hence, they were excluded. Also, the calendar time restrictions of the Multigeneration Register did not permit reliable identification of second‐generation relatives (e.g. grandparents). Finally, we restricted our analyses to first primary tumours. Therefore, 141 patients were excluded as they had a primary CRC diagnosis recorded in the Swedish Cancer Register, which predated the registration in the SCRCR. In case of metachronous tumours in the SCRCR, the first tumour recorded was kept. This information is visually represented in Fig. 1.

### Main measures

#### Family history

As in previous studies 10, 13, 14, 15, 17, a positive family history was defined as having at least one first‐degree relative (i.e. parent, sibling or child) affected with CRC. Relatives were only included in the definition if they had a diagnosis of colon or rectal adenocarcinoma as for cancers registered in the SCRCR. Cancer topography was defined using codes from the seventh edition of the International Classification of Disease (i.e. ICD7: 153–154), adopted in the National Cancer Register since 1958, whereas adenocarcinomas were identified using pathological anatomical diagnosis (PAD = 096).

Patients with potential Lynch syndrome were identified using a proxy definition and excluded in a sensitivity analysis. Patients were classified as possible Lynch if they had at least one additional Lynch‐related cancer or if they had a relative with CRC and a Lynch‐related cancer. Moreover, the Lynch‐related cancer had to precede the CRC diagnosis in the index patient. We included endometrial (ICD7: 172), ovarian (175.0 and 175.9), small bowel (152.0 and 152.9), stomach (151.0 and 151.9) and urinary (i.e. ureter: 181.1 or renal pelvis: 180.1) cancers in the definition of Lynch‐related cancers based on the literature that shows these cancers are more common in Lynch patients 29.

#### Stage

Pathological TNM stage [tumour size (T), lymph node involvement (N) and metastatic (M) status] was extracted from the SCRCR. We supplemented pathological information with information from clinical reports on metastasis (M) when this information was not available from the pathological report.

### Statistical methods

The distribution of patients' demographic and clinical characteristics is summarized for all patients by (positive and negative) family history and clinical characteristics including stage and grade. Using ordinal regression, we also assessed whether stage distribution varied by family history and age by regressing stage (0–IV) on the family history, age and their interaction using ordinal logistic regression.

The primary end‐points were relative survival and disease‐free survival (DFS). Relative survival is defined as the observed all‐cause survival amongst the patients divided by the expected survival in the general population (assumed free from the cancer in question). This provides an estimate of the excess mortality associated with a diagnosis of CRC 30. The expected survival in the general population was obtained from the Human Mortality Database and matched to our data using age, sex and calendar year 31. Patients were followed from their date of diagnosis until death, migration or end of study (31 December 2016), whichever happened first. For DFS, patients were followed from their date of diagnosis until relapse (i.e. distant or locoregional recurrence), death, migration or end of study (31 December 2016), whichever happened first. Patients were not included in the DFS if they had advanced disease (stage IV) as recorded in the SCRCR (*n* = 5346).

Flexible parametric survival models (FPMs) 32 adapted for relative survival were used to estimate excess mortality rate ratios (EMRR) and 95% confidence intervals (CI), comparing patients with positive and negative family history. Cumulative relative survival graphs were produced to visually represent any difference in prognosis between risk groups. For DFS, corresponding survival curves were estimated using the Kaplan–Meier method using standard FPM models. All regression models were adjusted for age at diagnosis (Model 1) as well as age and cancer stage (Model 2). The cumulative baseline hazard function in the FPM was modelled using a restricted cubic spline with four degrees of freedom. Due to the correlated structure of the data (i.e. within family groups), a robust sandwich estimator of the variance was applied to account for the clustering.

To determine whether the impact of family history on prognosis was modified by age at diagnosis (i.e. <50, 50–74 and 75+) or cancer location (i.e. rectum vs. colon), we included an interaction term between family history and age (or location) in our models. The Wald test was used to formally assess these interaction terms.

Primary analyses were based on individuals who had available information on stage in the SCRCR (i.e. complete cases). As part of our sensitivity analyses, we compared results based on complete cases with those obtained after imputing missing stage information either using stage from the Swedish Cancer Register or multiple imputation by chained equation based on the Nelson–Aalen estimate of cumulative hazard 33, 34, 35. The multiple imputation model included the main variables of our survival analyses together with auxiliary variables associated with pathological stage. These variables were tumour location, clinical stage, distant metastasis and vital status. Fifty imputation data sets were generated. Further sensitivity analyses were conducted to assess the robustness of our main findings when modifying the definition of family history, including half‐siblings and using a time‐dependent approach. The latter allowed us to define family history so that patients were only classified as having a positive family history if their diagnosis followed the one of a relative. This implies that the index patient was classified as having a negative family history if they had the earliest diagnosis within their family. This approach allowed us to assess whether behavioural factors may explain the association between family history and prognosis as individuals with a known family history may be more likely to seek opportunistic screening and adopt preventive measures. All analyses were conducted in Stata 14 36. The Stata STPM2 module was used to estimate flexible parametric survival models 37.

The Regional Ethics Board approved the study and waived the need for consent in view of the register‐based approach.

Data are available on request from the Swedish Colorectal Cancer Registry and Swedish Cancer Register.

## Results

There were 31 801 CRC patients who met our inclusion criteria (Fig. 1). Amongst the included patients, 10 407 (33%) deaths occurred during a median follow‐up of 2.8 years (range: 0–10). Overall, the median age at diagnosis was 68 years (interquartile range: 61–73). Fifty‐six per cent of all patients were men, and 5172 (16%) had a positive family history (Table 1). Patients with positive and negative family history had very similar clinical characteristics including stage distribution and tumour grade (Table 1). Treatments including abdominal resection and adjuvant chemotherapy were also equally distributed. Information on stage was missing for 4319 patients (14%).

**Table 1:** Clinical characteristics of 31 801 colorectal cancer (CRC) patients diagnosed in Sweden between 2007 and 2016 by family history of CRC

	All cases *N* = 31 801	Negative family history *N* = 26 629	Positive family history *N* = 5172
Sex			
Males	17 686 (56)	14 808 (56)	2878 (56)
Females	14 115 (44)	11 821 (44)	2294 (44)
Age			
<50	2181 (7)	1882 (7)	299 (6)
50–74	23 576 (74)	19 610 (74)	3966 (77)
75+	6044 (19)	5137 (19)	907 (18)
Median age (IQR)	68 (61–73)	68 (61–73)	67 (61–73)
Pathological stage^a^			
Stage 0	301 (1)	263 (1)	38 (1)
Stage I	4950 (16)	4126 (16)	824 (16)
Stage II	7944 (25)	6588 (25)	1356 (26)
Stage III	8948 (28)	7511 (28)	1437 (28)
Stage IV	5339 (14)	4464 (17)	875 (17)
Missing	4319 (14)	3677 (14)	642 (12)
Location			
Colon	20 497 (64)	17 048 (64)	3449 (67)
Rectum	11 304 (36)	9581 (36)	1723 (33)
Grade			
Low grade (high differentiation)	19 839 (62)	16 525 (62)	3314(64)
High grade (low differentiation)	5236 (17)	4418 (17)	818 (16)
Missing/not specified	6726 (21)	5686 (21)	1040 (20)
Abdominal resection surgery	*N* = 26 814	*N* = 22 426	*N* = 4388
Yes	25 333 (95)	21 139 (94)	4194 (96)
Adjuvant chemotherapy	*N* = 29 720	*N* = 24 864	*N* = 4856
Yes	10 430 (35)	8707 (35)	1723 (35)
Nonradical surgery	*N* = 27 127	*N* = 22 670	*N* = 4457
Yes	1169 (4)	1007 (4)	162 (4)

Results are provided as *n* (%) unless otherwise specified.

a Pathological TNM stage; tumour size (T), lymph node involvement (N) and metastatic status (M).

Table S1 shows the stage distribution in the complete cases compared to the imputed data. The proportion of stage IV cancers was higher in the imputed data which indicates more advanced cancers tend to have missing stage information in our sensitivity analyses. We found that the stage distribution varied by age and family history (*P* = 0.02). Specifically, young patients with a positive family history had more favourable stage distribution than their counterparts with a negative family history (OR = 0.71, 95% CI: 0.56–0.89, *P* = 0.004; Table 2, Table S2), whilst no difference was observed amongst the other age groups (*P* > 0.57). Amongst patients with a family history, we identified 272 (5.3%) patients who met our definition for Lynch and we refer to them as possible Lynch patients.

**Table 2:** Number of colorectal cancer cases (%) by pathological stage, age at diagnosis and family history amongst complete cases (*N* = 27 482), and odds ratios (OR), 95% confidence intervals (CI) and *P*‐values from ordinal regression exploring the association between family history and age group on stage (0–IV).

	Age <50 (*N* = 1929)			Age 50–74 (*N* = 20 534)			Age 75+ (*N* = 5019)		
	Positive family history (*N* = 1661)	Negative family history (*N* = 268)	OR (95% CI) *P*‐value	Positive family history (*N* = 17 046)	Negative family history (*N* = 3488)	OR (95% CI) *P*‐value	Positive family history (*N* = 4245)	Negative family history (*N* = 774)	OR (95% CI) *P*‐value
Stage 0	5 (2)	30 (2)	0.71 (0.56–0.89) 0.004	29 (1)	201 (1)	1.01 (0.94–1.1) 0.83	4 (1)	32 (1)	0.96 (0.84–1.1) 0.57
Stage I	43 (16)	243 (15)		625 (18)	3037 (18)		156 (20)	846 (20)	
Stage II	83 (31)	366 (22)		997 (29)	4792 (28)		276 (36)	1430 (34)	
Stage III	83 (31)	585 (35)		1119 (32)	5566 (33)		235 (30)	1360 (32)	
Stage IV	54 (20)	437 (26)		718 (21)	3450 (20)		103 (13)	577 (14)	

### Relative survival

**Figure 2:**
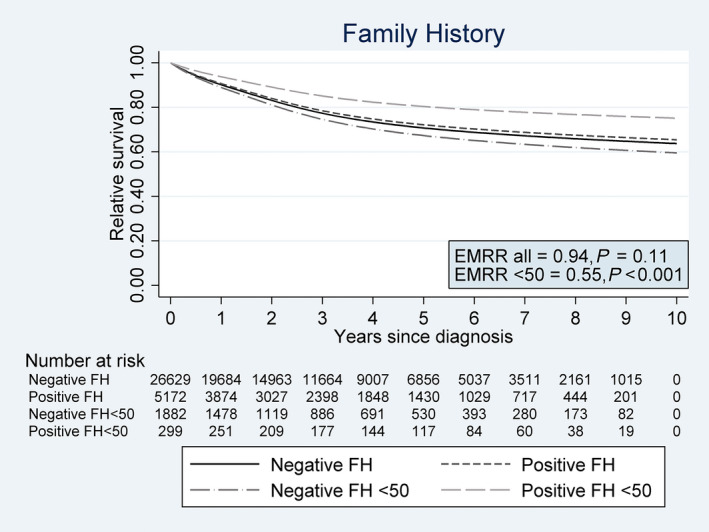
Relative survival for colorectal cancer patients with positive and negative family history (FH) of colorectal cancer overall and amongst patients <50 years of age at diagnosis. Excess mortality rate ratios (EMRR) for patients with positive family history versus those with negative family history are also presented in the figure.

Amongst patients with complete information for stage (*N* = 27 482), 8080 died (30%) during follow‐up. The 5‐year cumulative relative survival was 0.72 (95% CI: 0.71–0.74) and 0.71 (95% CI: 0.70–0.72) for individuals with a positive and negative family history, respectively (Fig. 2). Primary analyses showed family history was not associated with excess mortality when adjusting for age (EMRR = 0.94, 95% CI: 0.88–1.01, *P* = 0.11; Fig. 2) or age and stage (EMRR = 0.96, 95% CI: 0.89–1.03, *P* = 0.21; Table 3).

**Table 3:** Excess mortality rate ratios (EMRR) for relative survival and hazard ratios (HR), 95% confidence intervals (CI) and *P*‐values for disease‐free survival

Adjustment	Relative survival (positive FH vs. negative FH)		Disease‐free survival (positive FH vs. negative FH)	
	EMRR (95% CI)	*P*	HR (95% CI)	*P*
All patients				
Age	0.94 (0.88–1.00)	0.11	0.98 (0.91–1.05)	0.53
Age and stage	0.96 (0.89–1.03)	0.21	0.98 (0.91–1.06)	0.67
Young patients (<50)				
Age	0.55 (0.41–0.75)	<0.001	0.69 (0.47–1.0)	0.07
Age and stage	0.63 (0.47–0.84)	0.002	0.74 (0.50–1.1)	0.12

The estimates are obtained for patients with a positive family history (FH) compared to those with a negative history.

There was an interaction between family history and age (*P* = 0.002). Young (<50 years at diagnosis) patients with a positive family history had better prognosis than young patients with a negative family history (EMRR: 0.55, 95% CI: 0.41–0.75, *P* < 0.001) even after adjusting for age and stage (EMRR: 0.63, 95% CI: 0.47–0.84, *P* = 0.002). No difference was observed in the other age groups (*P* > 0.6).

There was an indication of an interaction between family history and location (colon vs. rectum, *P* = 0.054). We found that, for colon cancers, patients with a positive family history had lower excess mortality than those with a negative family history (EMRR: 0.89, 95% CI: 0.82–0.98, *P* = 0.01), but this association was weakened after adjusting for age and stage (EMRR: 0.95, 95% CI: 0.87–1.04, *P* = 0.26). No difference was observed between family risk group for rectal cancer when adjusting for age (EMRR: 1.04, 95% CI: 0.91–1.19, *P* = 0.53) or age and stage (EMRR: 0.96, 95% CI: 0.85–1.09, *P* = 0.56).

Our sensitivity analyses further showed that results were robust regardless of the exposure definition used in the overall sample as well as in the analyses amongst young patients (Tables S3 and S4). Indeed, we found that including half‐siblings and using a time‐dependent approach did not change the results. Similarly, the various approaches used to estimate missing data had little impact on the results. Lastly, we found that excluding possible Lynch patients did not alter our findings.

### Disease‐free survival

**Figure 3:**
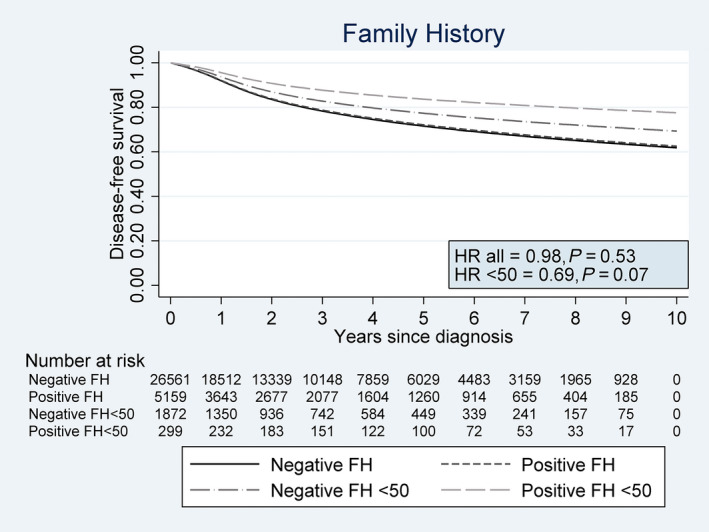
Disease‐free survival for colorectal cancer patients with positive and negative family history (FH) of colorectal cancer overall and amongst patients <50 years of age at diagnosis. Hazard ratios (HR) for patients with positive family history versus those with negative family history are presented in the figure.

Amongst the 22 138 patients with stage I–III disease at diagnosis, 5036 (23%) patients died or relapsed during follow‐up. Of these, 2869 (13%) relapsed either loco‐regionally and/or with distant metastases. Relapse rates were similar amongst patients with a negative (13.6%) and positive (14.5%) family history. No association was found between family history and DFS when adjusting for age (HR = 0.98, 95% CI: 0.91–1.05, *P* = 0.53; Fig. 3) or age and stage (HR = 0.98, 95% CI: 0.91–1.06, *P* = 0.67).

We found no evidence for an interaction effect between family history and age on DFS (*P* = 0.18) or cancer location (*P* = 0.60). However, given the evidence of an association with family history and relative survival amongst patients <50 years of age, we still performed exploratory analyses of family history and DFS in this subgroup. We found some evidence that young patients with a positive family history had an indication of a better prognosis than young patients with a negative family history even after adjusting for age and stage (Table 3; Fig. 3). Still, in our time‐dependent sensitivity analysis, we observed an improved prognosis on DFS amongst young patients with positive family history compared to their counterparts with negative history when adjusting for age and stage (HR = 0.65, 95% CI: 0.43–0.98, *P* = 0.04; Table S3). Family history had no effect on prognosis in older patients (*P* > 0.53). Further sensitivity analyses showed our main results were robust regardless of the definition of family history, missing data estimation or exclusion of possible Lynch patients (Tables S3 and S4).

## Discussion

This is, to our knowledge, the first study to explore the potential modifying role of age at diagnosis and cancer location on the association between family history and prognosis using population‐based data. We found that age at diagnosis modified this association as young patients (<50) with a positive family history had better relative survival than their counterparts. This association was weakened but did not disappear after adjusting for stage and age.

Better prognosis in young patients with a positive family history may be partly explained by our finding that they had less advanced cancers than their counterparts with a negative family history. Our results suggest that young patients who are aware of their family history may adopt healthy behaviours (e.g. opportunistic screening) and/or make healthy lifestyle changes, such as reducing smoking and red meat intake 38, 39, which improve their prognosis. This suggestion appears to be further supported by the results showing improved prognosis in disease‐free survival in young patients with family history compared to those with no family history in our time‐dependent analyses, that is when patients were classified as having a positive family history only if their diagnosis followed the one of a relative. However, these results need to be interpreted with caution as they are exploratory in nature. Information on health behaviours was not available in our data set and we cannot corroborate this hypothesis. Future studies are necessary to assess whether younger patients with a positive family history are more likely to adopt healthy behaviours once they become aware of being at risk of developing cancer due to their family history. However, since we observed a favourable outcome amongst young individuals even after adjustment for stage, opportunistic screening is likely not the only explanation for our findings.

Another possible explanation for improved relative survival and staging in young patients with a family history may be related to genetic differences. However, our results did not change after excluding possible Lynch patients, who have been found to have good prognosis 7, 8. Therefore, it is possible that behavioural changes in young patients outweigh any genetic predisposition. Future studies should aim to identify the mechanisms that may explain this link.

Our results are contradictory of a previous study which found worse prognosis in young patients with an affected sibling (vs. no sibling) 16. The difference in results may be driven by timing of diagnoses. It is possible that results by Slattery and colleagues (1995) are driven by the fact that patients with worse prognosis were the proband in the families or that the lapse between siblings' diagnoses was too short to allow any protective health‐conscious behaviour to improve prognosis of the sibling. In contrast, in our study, first‐degree relatives included also parents, and it is more likely that the parents' diagnoses precede the children's diagnoses and, hence, allow for positive behavioural changes. This also supports our hypothesis that being aware of family history leads to health‐aware behaviour that, with time, may affect tumour evolution 38, 39.

A few previous studies have not observed an overall association between family history and prognosis 9, 10, 11. Two of these studies are also based on population‐based data from the Swedish Cancer Register. However, these studies investigated overall survival and did not explore the modifying effect of age. Moreover, studies which found an association between family history and prognosis were smaller and relied on self‐reported family history 15, 16, 17. A major advantage of our study is that, using population data from the SCRCR, we were able to explore the modifying role of age, stage and cancer site on the association between family history and relative survival as well as disease‐free survival.

We also observed some evidence of a modifying role of cancer location on the association between family history and relative survival. Patients with a positive family history had lower excess mortality than their counterparts for colon but not for rectal cancers. However, after adjusting for stage, the protective effect of family history on colon cancer patients was weakened. Previous studies found that a positive family history was associated with better overall survival for colon but not rectal cancers even after adjusting for a number of cancer characteristics including stage 15, 17. These somewhat differing results may be due in part to methodological differences as previous studies were based on smaller cohorts and explored overall survival rather than relative or disease‐free survival.

Sensitivity analyses indicated that our findings were robust and did not vary depending on the approach used to deal with missing information for stage or the definition used for the family history. It is particularly interesting that including half‐siblings in our definition of family history did not alter the main results. Therefore, half‐siblings could potentially be included when defining family history to identify high‐risk individuals.

A limitation of the study is that no genetic information was available so we could not identify patients with Lynch syndrome. However, we identified patients with suspected Lynch using a proxy definition based on the diagnosis of Lynch‐related cancers in either patients or their relatives. Excluding patients who met these criteria did not change our results, which suggests better prognosis in individuals with family history is not driven by possible Lynch patients in our sample. A further limitation is that we may not have captured a full cancer family history as information was only available for the years after the start of registration of relatives (i.e. left truncation). Lastly, we would have ideally liked to use the Amsterdam criteria 40 to identify potential patients with Lynch syndrome; however, it was not possible to reconstruct family trees from the Multigeneration Register for more than two generations. Nonetheless, the definition of family history as having at least 1 first‐degree relative is associated with a 2‐ to 4‐fold increased risk of developing CRC 6 and widely used in research 13, 14, 15. The main strength of the study is the clinical richness and near complete coverage of the data. Moreover, family history is based on the linkage with population‐level register data rather than self‐report, which may not be as accurate.

In conclusion, a positive family history was found to be linked with good prognosis in CRC patients diagnosed before the age of 50, but not amongst all patients. In particular, we observed better (relative) survival in young patients with a positive family history compared to those with a negative one. This may be partly due to the fact these patients are more likely to adopt preventive measures (e.g. attend screening) and/or healthy lifestyle changes because of increased awareness. Stage did not fully explain this association so further exploration is required to understand potential mechanisms including lifestyle changes that may underlie improved prognosis amongst young patients and that could then be applied more broadly.

## Conflict of interest

No conflict of interest.

## Supporting information


**Table S1.** Distribution of pathological stage in the available data in Swedish Colorectal Cancer Registry (SCRCR; complete cases) and using different methods to estimate missing information.
**Table S2.** Clinical characteristics of young (<50) colorectal cancer (CRC) patients diagnosed in Sweden between 2007 and 2016 by family history (*N* = 2181).
**Table S3.** Sensitivity analyses results.
**Table S4.** Subgroup sensitivity analyses: Relative survival by location.
