# Supplementary material for: Family history of colorectal cancer and survival: a Swedish population‐based study
Source: J Intern Med. 2020 Mar 3;287(6):723–33. doi: 10.1111/joim.13036 (PMC7318575; doi:10.1111/joim.13036)
Supplement: Supplementary file 1 — Table S1. Distribution of pathological stage in the available data in Swedish Colorectal Cancer Registry (SCRCR; complete cases) and using different methods to estimate missing information. Table S2. Clinical characteristics of young (<50) colorectal cancer (CRC) patients diagnosed in Sweden between 2007 and 2016 by family history (N = 2181). Table S3. Sensitivity analyses results. Table S4. Subgroup sensitivity analyses: Relative survival by location. [file JOIM-287-723-s001.docx]

**Supplementary Table 1. Distribution of pathological stage in the available data in SCRCR (complete cases) and using different methods to estimate missing information**

|  | **Complete cases*** | **Imputed cases**** | **MI imputed cases***** |
| --- | --- | --- | --- |
|  | **N (%)** | **N (%)** | **% (95% CI)** |
| **Stage 0** | 301 (1.0) | 301 (1.0) | 1.1 (1.08- 1.11) |
| **Stage I** | 4,950 (15.6) | 5,056 (17.2) | 17.5 (17.3- 17.5) |
| **Stage II** | 7,944 (25.0) | 8,033 (25.2) | 27.0 (26.9- 27.1) |
| **Stage III** | 8,948 (28.2) | 9,204 (28.9) | 31.0 (30.8- 31.1) |
| **Stage IV** | 5,339 (13.6) | 6,867 (21.6) | 23.5 (23.4-23.6) |
| ***Missing*** | *4,319 (13.6)* | *2,340 (7.4)* | *n/a* |

*N (%) for complete cases in SCRCR

**N (%) for complete cases in SCRCR complemented using stage information from the Swedish Cancer Register

*** % (95%CIs) across the datasets obtained using multiple imputation

**Supplementary Table 2.** Clinical characteristics of young (<50) CRC patients diagnosed in Sweden between 2007 and 2016 by family history (N= 2181). Results are provided as n (%) unless otherwise specified.

|  | **All cases**  **N = 2,181** | **Negative family history**  **N=1,882** | **Positive family history**  **N=299** | |
| --- | --- | --- | --- | --- |
| **Sex** |  |  | |  |
| Males | 1,191 (55) | 1,023 (54) | | 168 (56) |
| Females | 990 (45) | 859 (46) | | 131 (44) |
| **Median age (IQR)** | 44 (39-47) | 44 (39-47) | | 44 (40-47) |
| **Pathological Stage** |  |  | |  |
| Stage 0 | 35 (2) | 30 (2) | | 5 (2) |
| Stage I | 286 (13) | 243 (13) | | 43 (14) |
| Stage II | 449 (21) | 366 (20) | | 83 (28) |
| Stage III | 668 (31) | 585 (31) | | 83 (28) |
| Stage IV | 491 (23) | 437 (23) | | 54 (18) |
| Missing | 252 (12) | 221 (12) | | 31 (10) |
| **Location** |  |  | |  |
| Colon | 1,373 (63) | 1,177 (63) | | 196 (66) |
| Rectum | 808 (37) | 705 (37) | | 103 (34) |
| **Grade** |  |  | |  |
| Low grade  (high differentiation) | 1,272 (58) | 1,089 (58) | | 183 (61) |
| High grade  (low differentiation) | 415 (19) | 351 (19) | | 64 (22) |
| Missing/not specified | 494 (23) | 442 (23) | | 52 (17) |
| **Abdominal resection surgery** | **N =1,873** | **N=1,610** | | **N=263** |
| Yes | 1,768 (94) | 1,513 (94) | | 255 (97) |
| **Adjuvant chemotherapy** | **N = 2,033** | **N = 1,747** | | **N = 286** |
| Yes | 1,042 (51) | 901 (52) | | 141 (49) |
| **Non-radical surgery** | **N=1,687** | **N=1,603** | | **N = 264** |
|  | 98 (5) | 95 (6) | | 3 (1) |

^$^ Pathological TNM stage; tumour size (T), lymph node involvement (N) and metastatic status (M).

**Supplementary Table 3.** Sensitivity analyses results. Excess mortality rate ratios (EMRR) and hazard ratios (HR) for disease-free survival. The estimates are obtained for patients with a positive family history compared to those with a negative history.

|  |  | **Relative survival** | | **Disease-free survival** | | |
| --- | --- | --- | --- | --- | --- | --- |
|  | **Adjustment** | **EMRR (95%CI)** | **p** | **HR (95%CI)** | **p** | |
| **Exposure is time dependent^^^** | **Age** | 0.93 (0.86-1.01) | 0.06 | 0.96 (0.89- 1.04) | 0.36 | |
|  | **Age and stage** | 0.95 (0.89-1.03) | 0.22 | 0.98 (0.90-1.06) | 0.53 | |
| **Include half siblings in the definition of family history** | **Age** | 0.94 (0.87-1.01) | 0.09 | 0.97 (0.90-1.04) | 0.36 | |
|  | **Age and stage** | 0.95 (0.88-1.02) | 0.16 | 0.97 (0.90-1.05) | 0.47 | |
| **Impute missing stage information using SCR stage¥** | **Age** | 0.92 (0.86-0.98) | 0.02 | 0.97 (0.90-1.05) | 0.40 | |
|  | **Age and stage** | 0.94 (0.88-1.00) | 0.07 | 0.98 (0.91-1.05) | 0.52 | |
| **Impute missing stage information via Multiple Imputation by chained equation^$^** | **Age** | 0.92 (0.86-0.98) | 0.01 | 0.97 (0.90-1.04) | 0.32 | |
|  | **Age and stage** | 0.94 (0.88-1.01) | 0.06 | 0.97 (0.91-1.05) | 0.44 | |
| **Lynch-like patients are removed^#^** | **Age** | 0.96 (0.89-1.0) | 0.30 | 0.99 (0.92-1.1) | 0.79 | |
|  | **Age and stage** | 0.96 (0.90-1.0) | 0.32 | 0.99 (0.92-1.1) | 0.83 | |
| **Young patients (<50)** | | | |  | | |
|  |  | **Relative survival** | | **Disease-free survival** | | |
|  | **Adjustment** | **EMRR (95%CI)** | **p** | **HR** | | **P** |
| **Exposure is time dependent^** | **Age** | 0.54 (0.39-0.75) | <0.001 | 0.62 (0.41-0.95) | | 0.03 |
|  | **Age and stage** | 0.64 (0.46-0.88) | 0.01 | 0.65 (0.43-0.98) | | 0.04 |
| **Include half siblings in the definition of family history** | **Age** | 0.57 (0.42-0.76) | <0.001 | 0.68 (0.46-1.0) | | 0.05 |
|  | **Age and stage** | 0.64 (0.48-0.85) | 0.002 | 0.72 (0.49-1.1) | | 0.09 |
| **Impute missing stage information using SCR stage¥** | **Age** | 0.58 (0.44-0.77) | <0.001 | 0.74 (0.51-1.1) | | 0.12 |
|  | **Age and stage** | 0.66 (.50 to .87) | 0.003 | 0.78 (0.54-1.1) | | 0.20 |
| **Impute missing stage information via Multiple Imputation by chained equation^$^** | **Age** | 0.61 (0.47-0.79) | <0.001 | 0.72 (0.50-1.04) | | 0.08 |
|  | **Age and stage** | 0.70 (0.54-0.91) | 0.01 | 0.77 (0.54-1.11) | | 0.17 |
| **Lynch-like patients are removed^#^** | **Age** | 0.60 (0.44-0.81) | 0.001 | 0.75 (0.50-1.1) | | 0.15 |
|  | **Age and stage** | 0.66 (0.49-0.88) | 0.005 | 0.78 (0.53-1.1) | | 0.20 |

^^^ In the time dependent approach, patients are only be classified as having a positive family history if their diagnosis followed the one of a relative.

^¥^ RS= 29461; DFS = 22,589

^$^ RS: 31801; DFS: sample size varies between imputations between 24,201 and 24,297

^#^ Patients were defined Lynch-like if they had a Lynch-related cancer or if they have a relative with CRC and a Lynch-related cancer which preceded the CRC diagnosis in the index patient. Lynch-related cancers are: endometrial (icd7: 172), ovarian (175.0 and 175.9), small bowel (152.0 and 152.9), stomach (151.0 and 151.9), and urinary (i.e. ureter: 181.1 or renal pelvis: 180.1). RS: 27,247; DFS: 21,938

**Supplementary Table 4.** Subgroup sensitivity analyses: Relative survival by location. The estimates are obtained for patients with a positive family history compared to those with a negative history.

|  | **Relative survival** | | | | |
| --- | --- | --- | --- | --- | --- |
|  |  | **Colon** | | **Rectum** | |
|  | **Adjustment** | **EMRR (95%CI)** | **p** | **EMRR (95%CI)** | **p** |
| **Main** | **Age** | 0.89 (0.82-0.98) | 0.01 | 1.04 (0.91- 1.19) | .53 |
|  | **Age and stage** | 0.95 (0.87-1.04) | 0.26 | 0.96 (0.85 -1.09) | .56 |
| **Exposure is time dependent^^^** | **Age** | 0.89 (0.82-0.98) | 0.01 | 1.04 (0.92- 1.19) | .53 |
|  | **Age and stage** | 0.95 (0.86-1.04) | 0.25 | 0.97 (0.85- 1.10) | 0.60 |
| **Include half siblings in the definition of family history** | **Age** | 0.89 (0.82-0.99) | 0.01 | 1.02 (0.90-1.17) | 0.71 |
|  | **Age and stage** | 0.95 (0.87-1.03) | 0.24 | 0.95 (0.84- 1.08) | 0.42 |
| **Impute missing stage information using SCR stage¥** | **Age** | 0.88 (0.81-0.96) | 0.002 | 0.99 (0.88- 1.12) | 0.92 |
|  | **Age and stage** | 0.93 (0.86-1.01) | 0.09 | 0.94 (0.84- 1.06) | 0.31 |
| **Impute missing stage information via Multiple Imputation by chained equation^$^** | **Age** | 0.88 (0.81-0.95) | 0.001 | 1.00 (0.90-1.12) | 0.99 |
|  | **Age and stage** | 0.94 (0.87-1.01) | 0.10 | 0.95 (0.85- 1.06) | 0.34 |
| **Lynch-like patients are removed^#^** | **Age** | 0.90 (0.83-0.99) | 0.37 | 1.07 (0.94- 1.22) | 0.33 |
|  | **Age and stage** | 0.96 (0.88- 1.05) | 0.35 | 0.97 (0.86-1.11) | 0.69 |
|  | **Disease-Free Survival** | | | | |
| **Main** | **Age** | 0.96 (0.88-1.06) | 0.41 | 1.00 (0.88-1.14) | 0.95 |
|  | **Age and stage** | 0.97 (0.87-1.07) | 0.54 | 1.02 (0.90-1.16) | 0.78 |
| **Exposure is time dependent^^^** | **Age** | 0.96 (0.87-1.05) | 0.34 | 0.99 (0.86- 1.13) | 0.85 |
|  | **Age and stage** | 0.97 (0.88-1.07) | 0.52 | 1.00 (0.88- 1.15) | 0.97 |
| **Include half siblings in the definition of family history** | **Age** | 0.95 (0.87-1.04) | 0.26 | 1.00 (0.88- 1.14) | 0.95 |
|  | **Age and stage** | 0.95 (0.87-1.04) | 0.31 | 1.02 (0.90- 1.16) | 0.76 |
| **Impute missing stage information using SCR stage¥** | **Age** | 0.96 (0.88-1.05) | 0.36 | 1.00 (0.88- 1.13) | 0.98 |
|  | **Age and stage** | 0.97 (0.89-1.06) | 0.49 | 1.01 (0.89- 1.15) | 0.88 |
| **Impute missing stage information via Multiple Imputation by chained equation^$^** | **Age** | 0.95 (0.87-1.03) | 0.22 | 1.01 (0.90- 1.13) | 0.91 |
|  | **Age and stage** | 0.96 (0.87-1.04) | 0.31 | 1.02 (0.91- 1.15) | 0.74 |
| **Lynch-like patients are removed^#^** | **Age** | 0.97 (0.89-1.07) | 0.57 | 1.02 (0.90- 1.17) | 0.73 |
|  | **Age and stage** | 0.98 (0.89-1.08) | 0.65 | 1.03 (0.90-1.17) | 0.67 |

^^^ In the time dependent approach, patients are only be classified as having a positive family history if their diagnosis followed the one of a relative.

^¥^ RS= 29461; DFS = 22,589

^$^ RS: 31801; DFS: sample size varies between imputations between 24,201 and 24,297

^#^ patients were defined Lynch-like if they had a Lynch-related cancer or if they have a relative with CRC and a Lynch-related cancer which preceded the CRC diagnosis in the index patient. Lynch-related cancers are: endometrial (icd7: 172), ovarian (175.0 and 175.9), small bowel (152.0 and 152.9), stomach (151.0 and 151.9), and urinary (i.e. ureter: 181.1 or renal pelvis: 180.1). RS: 27,247; DFS: 21,938
